# Repair of Large Lip Vermilion defects with Mutual Cross Lip Musculomucosal Flaps

**Published:** 2012-01

**Authors:** Ali Manafi, Mohammad Ahmadi Moghadam, Maryam Mansouri, Hamed Bateni, Mahnaz Arshad

**Affiliations:** 1 Department of Plastic Surgery, Tehran University of Medical Sciences, Tehran, Iran;; 2 Plastic Surgeon, Day Hospital, Tehran, Iran;; 3 Plastic Surgeon, Qom, Iran;; 4 Plastic Surgeon, Mehr Hospital, Tehran, Iran;; 5 Department of Prosthodontics, Tehran University of Medical Sciences, Tehran, Iran

**Keywords:** Vermilion border, Flap, Cleft lip, Mutual cross-lip musculomucosal flaps

## Abstract

**BACKGROUND:**

Vermilion irregularities are common secondary deformities after cleft lip repair, regressed or resected hemangiomas, trauma and tumor surgeries. Vermilion deficiency attracts considerable attention and detracts from an otherwise excellent lip repair. Minor and moderate vermilion defects can be corrected with upper lip advancement, rotation flaps, tongue flaps or grafts. Major defects defy correction with local flaps. A technique is described for correction of large absolute tissue defects of the vermilion using Mutual Cross-Lip Musculomucosal Flaps (MCLMF) Or Ahmad-Ali's flaps.

**METHODS:**

This technique was applied in eight patients with major vermilion defects secondary to hemangioma regression, neoplasia, and trauma. Reconstruction with MCLMF led to create a balanced donor and recipient lips appearance and function.

**RESULTS:**

There were no postoperative complications. Surgical results were satisfactory in all patients, and sufficient lip mobility with adequate bulk was maintained. One patient demonstrated minimal transient lip tightening.

**CONCLUSION:**

Use of Ahmad-Ali's flaps in selected patients resulted in successful reconstruction of severe vermilion defects.

## INTRODUCTION

Vermilion border irregularities are common secondary deformities following cleft lip repair. As a matter of fact, irregularities of the vermilion cause undesirable attention with adverse social consequences.^1^ The goal of surgery is to restore both the function and appearance of the lip. This can usually be achieved with local flaps.^2^

Several methods are available to correct vermilion defect including Z-plasties, V-Y advancement, transposition flaps, free grafts, and cross lip flaps. Misalignment of the vermilion border, either minor or moderate, can be repaired by a V-Y advancement, Z-plasty technique or advancement of lip mucosa.^3^^-^^5^

Several procedures aiming to fill large vermilion gaps have been described in the literature. The donor sites of interest are: i) Remaining vermilion and mucosa of defective lip; ii) Tongue; and iii) Mucosa that covers the inner surface of normal lip.^3^ In this study, we propose a novel method that has advantages over the above mentioned methods.

## MATERIALS AND METHODS

*Surgical Techniques*

There were eight patients who have gone through the proposed treatment procedure for lip defects from November 2007 to August 2011. To start the procedure, the patients were notified that it might require 10 to 14 days of lips closure. General anesthesia was obtained by nasal intubation with nasogastric tube. This helped patient's gastric decompression. 

**Fig. 1:**
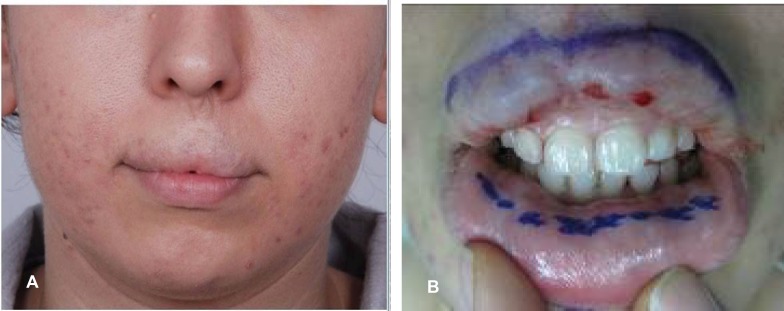
Preoperative markings

**Fig. 2:**
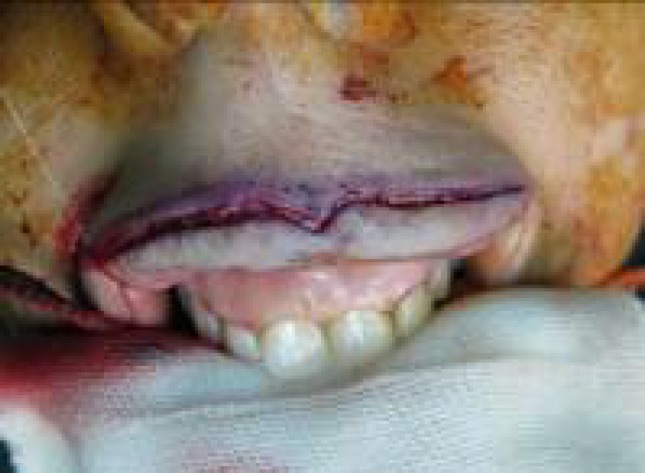
Incision and flap preparation of upper lip to prepare the bed for transfer of the cross lip flap

An incision at the border of vermilion-skin interface was performed that dissected lip mucosa and superficial layers of orbicularis oris muscle, with a thickness of about 1.5-2 mm. This flap was dissected about 1.5 cm from skin to wet vermilion and tapered to the lip commissure. An important consideration was to make suturing line invisible and positioned towards mouth (Figure 1 and 2).

**Fig. 3:**
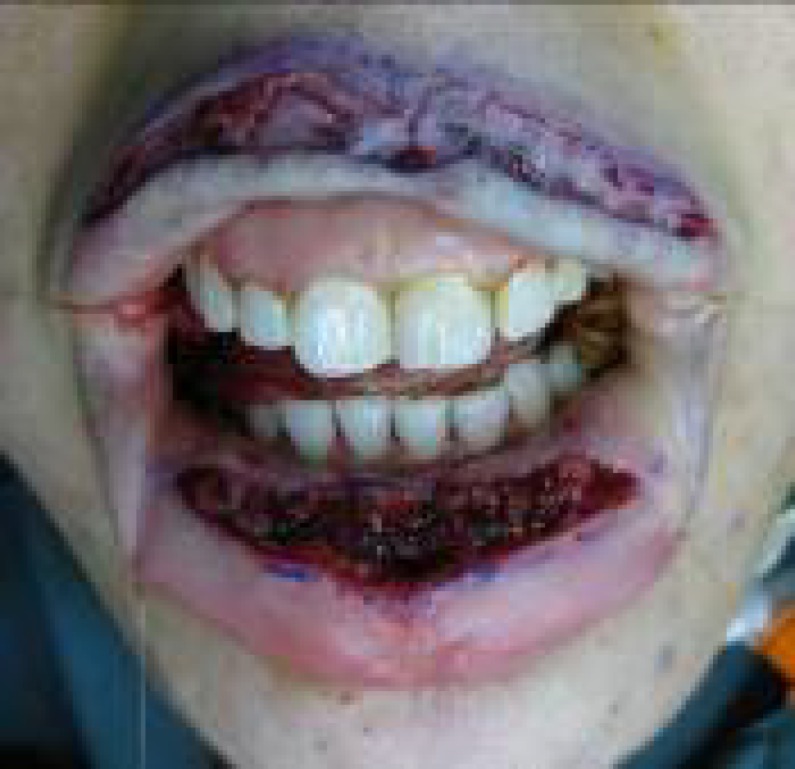
Both flaps are ready to transfer.

An incision was performed over the mucosa of the opposite normal lip with the same dimensions as that of the defective lip (flap in oral mucosa of the lip). This was done in such a way that the sutures at donor side to be invisible in repose or speaking situations after cutting and inserting of the flap. This time, musculomucosal flap dissection was performed at the depth of vestibule towards outside with 1.5 to 2 mm thickness (Figures 3).

**Fig. 4:**
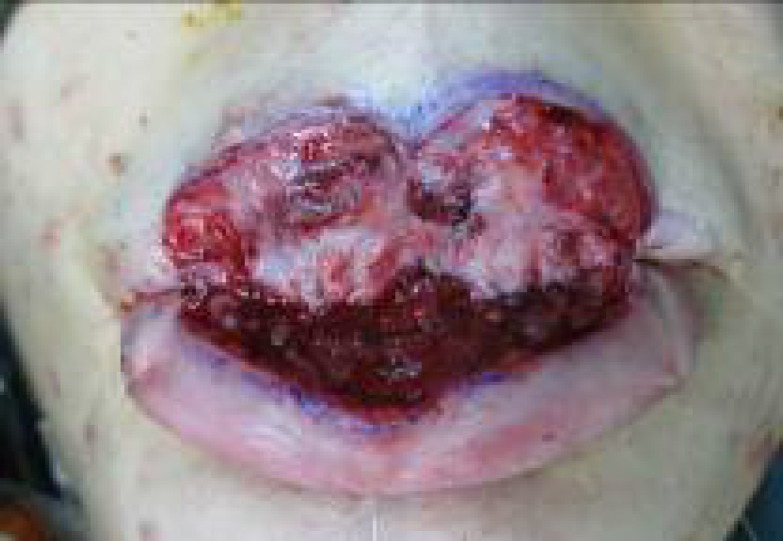
The defective lip flap sutured the bed of the donor flap.

**Fig. 5:**
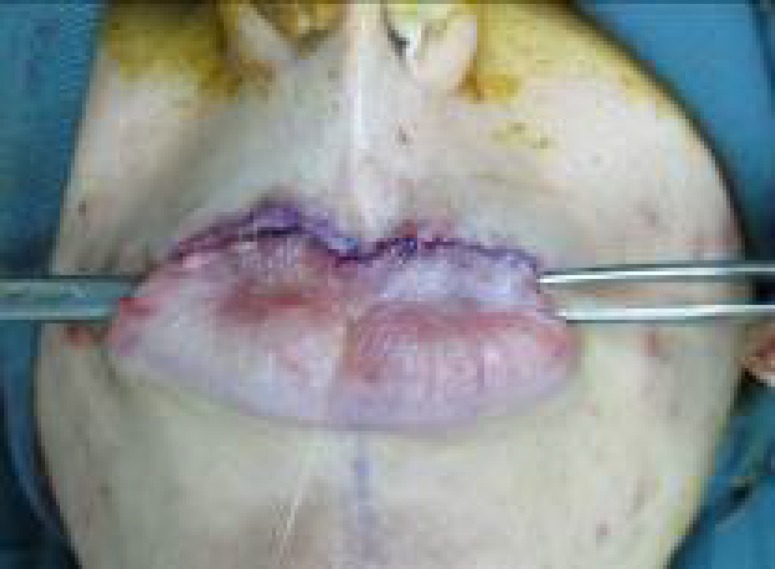
Donor flap sutured to the recipient site (upper lip).

**Fig. 6:**
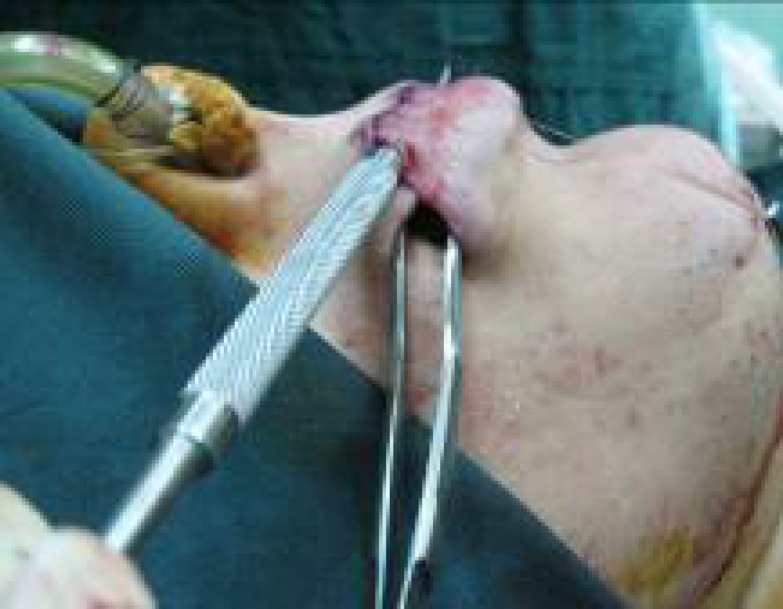
Both flaps were insected to be transferred. Each surgical instrument was inserted underneath of each flap

Then, defective lip flap edge was sutured to the opposite lip mucosa donor defect edge near the depth of vestibule with vicryl 4.0 or 5.0 (Figure 4). Next, the normal lip flap was directly sutured to the edge of the skin of the defective lip (Figure 5). Therefore, in the proposed method the defective side was repaired by a flap from the normal side. In this method, the donor site of the donor lip was repaired by diseased mucosa of the defective lip mutually, which used to be discarded in other methods. This was as if a lining flap and a coverage flap are placed on each other. In Figure 6, a surgical instrument was cross-passed underneath the two flaps and another instrument was passed between the two flaps to help better understanding of the procedure. In total or near total situations, both mouth commissures were open, which could be used for patient's hygiene and nutrition purposes.

**Fig. 7:**
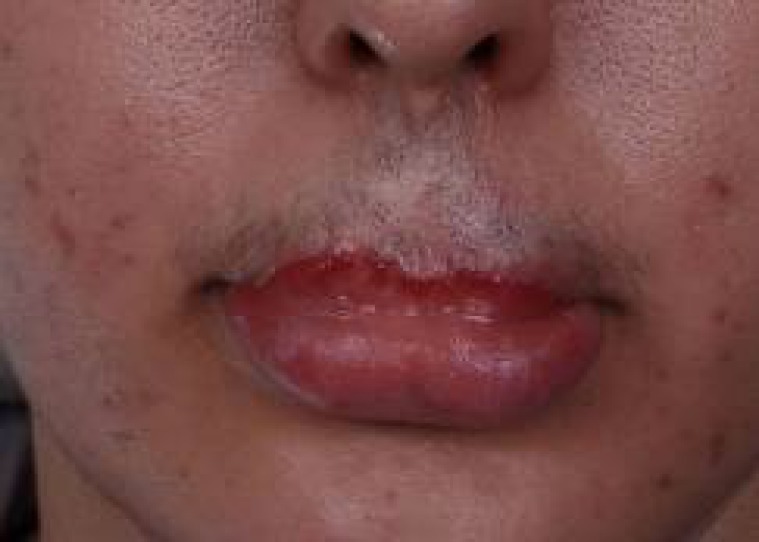
Wound healing of flaps are acceptable and the patient is ready to divide the flap pedicles and to open the mouth.

**Fig. 8:**
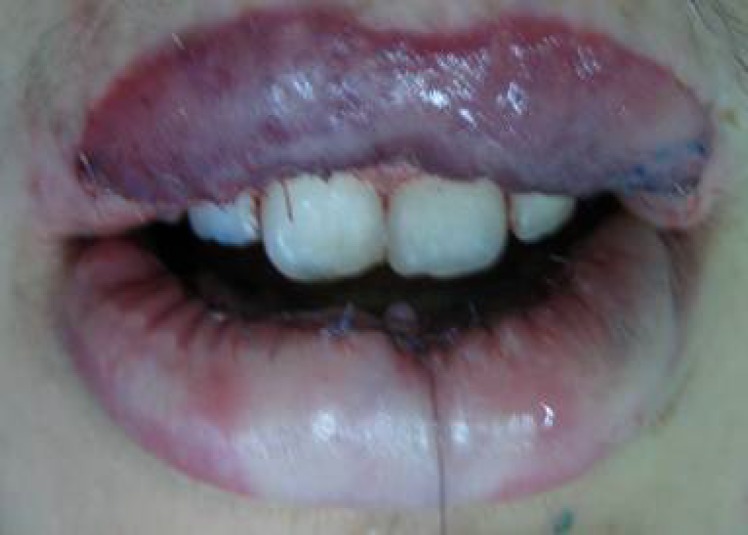
The pedicles were divided and complete inset of flaps were performed

**Fig. 9:**
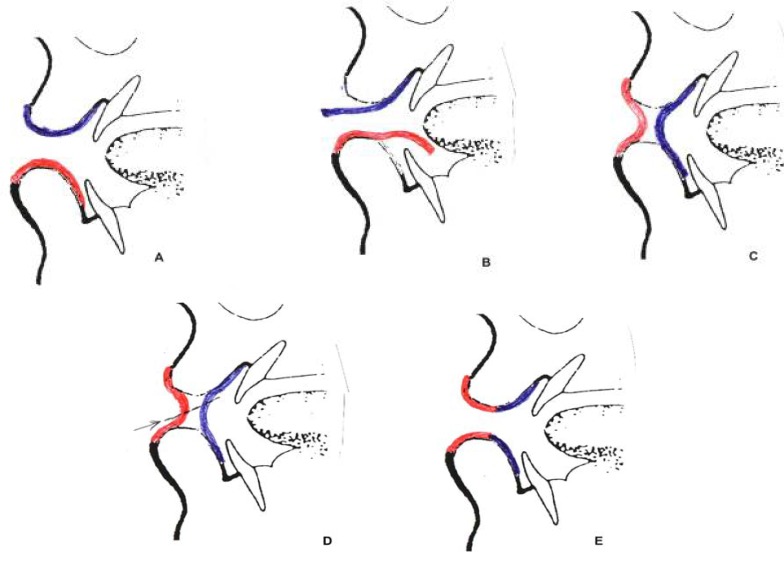
Schematic presentation of mutual cross lip flaps transfer: a: Upper lip is pathologic (dark blue) and lower lip is normal (Red), b: Both lips’ flaps were dissected, c: Both flaps were inserted, d: Beveled flaps’ pedicles division, e: Complete inset of flaps, both lips have normal looking verrmilion (Red).

Then the patient was extubated and N/G tube was removed and topical antibiotic was applied on the lips. A second operation was performed to dissect each flap from its pedicle after 10-14 days (Figure 7). This operation was performed under deep sedation. Note that the first cut was done on coverage flap. Then the mucosa between the two lips was dissected under a beveled cut. Then inserting of flaps was completed and suturing of the flaps was performed (Figure 8). Schematic presentation of whole procedure is provided in Figure 9.

**Fig. 10:**
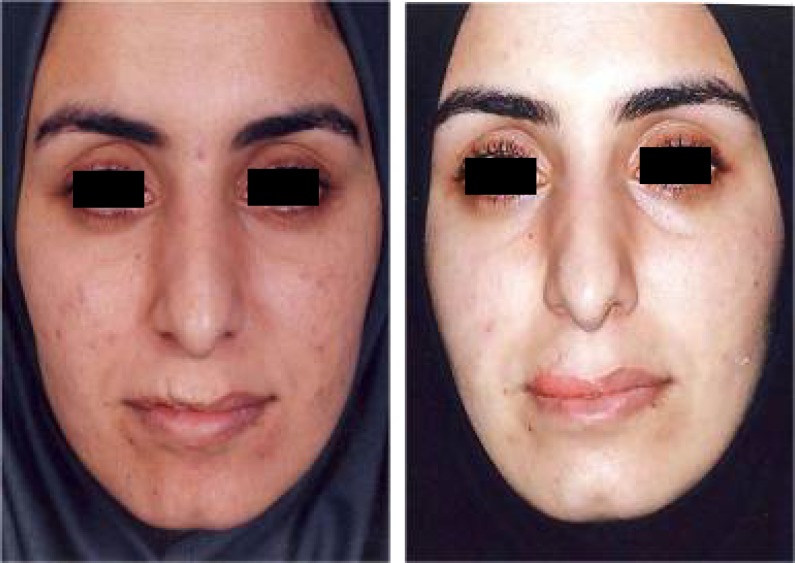
A: Right side of upper lip is defective. B: the defect has been corrected by MCLMF transfer

**Fig. 11:**
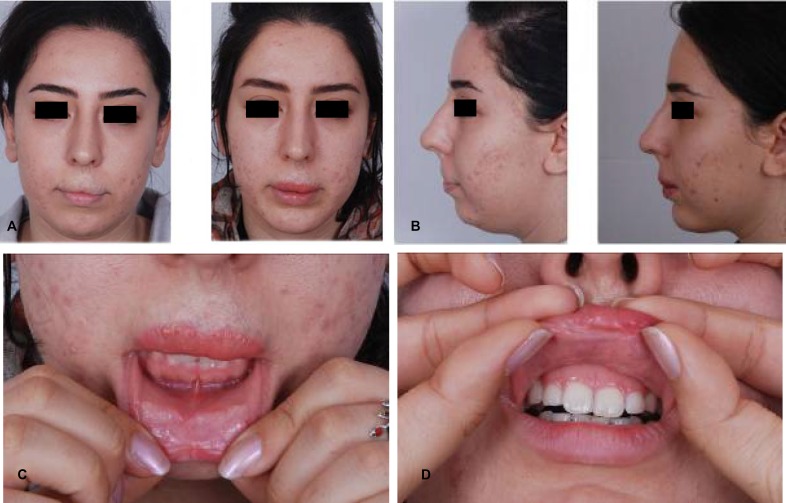
Upper lip regressed hemongioma with retrogenia. The problem was improved by MCMLF transfer and sliding advancement genioplasty. A: Frontal views (Right: Preoperative view. Left: Post operative view). B: Profile views. C: Inner side of lower lip shows insertion of defective lip flap surrounded by normal mucosa. d: Transition of transferred normal looking mucosa to abnormal pale mucosa of upper lip

**Fig. 12:**
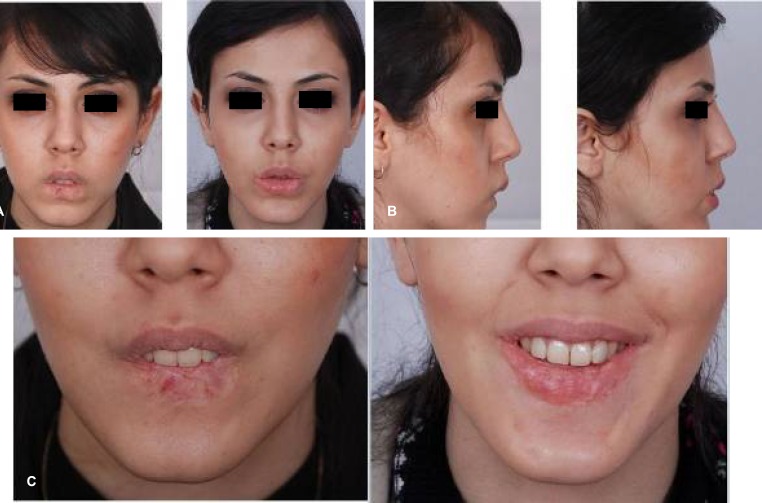
Lower lip regressed and resected hemangioma, MCLMF transfer, anterior sliding genioplasty and lower lip dermograft. A: Frontal views. B: Profile views. C: Smiling close up views.

**Fig. 13:**
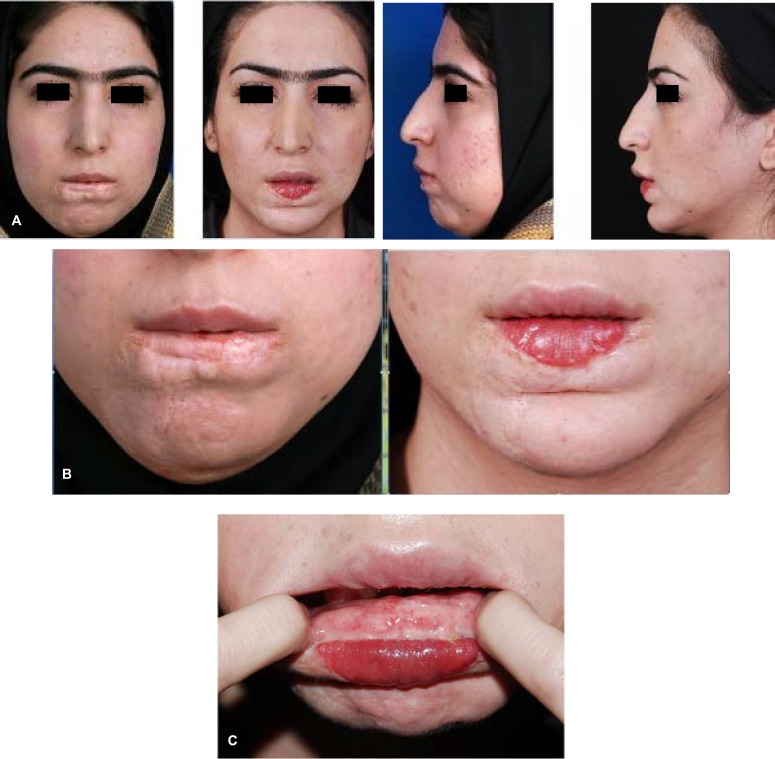
(A-C) Regressed and resected lower lip hemangioma, MCLMF transfer and anterior sliding genioplasty were carried out. A: Frontal views. B: Close up views. C: Gross difference between transferred flap and recipient bed colors.

After cutting pedicles of the flaps, good oral hygiene should be maintained. The patient was encouraged to keep the new lips moist for several weeks as they were exposed to the air and sun. This surgical operation can be performed individually or in combination with other operations. As it can be seen in our cases, in three of them the operations were done along with genioplasty surgery and in one case in combination with fat injection (Figures 10-13).

*Case Series*

The technique is illustrated in eight patients with severe lip vermilion defects. Each patient was under mutual cross- lip musculomucosal switch flaps (MCLMF) surgery.

Case 1 was a 34-year-old female with hemangioma excision. Affected area was on the right side of upper lip. This case was the first patient in this series, which was performed by A Moghaddam. Patient satisfaction was very good (Figure 10).

Case 2 was a 25-year-old female with hemangioma. Affected area was near the entire upper lip vermilion. Anterior sliding genioplasty was also performed for the patient. Patient satisfaction was very good (Figure 11). 

Case 3 was a 24-year-old female with hemangioma excision. Affected area was the entire lower lip vermilion. Anterior sliding genioplasty, facial lipoplasty and dermofat grafting of the lower lip also was performed for the patient. Patient satisfaction was excellent (Figure 12).

Case 4 was a 26-year-old female with hemangioma. Affected area was the entire lower lip vermilion and right side of gonion. MCLMF with genioplasty was done .She was satisfied with good result. She needed a minor lip revision procedure for better lip adjustment (Figure 13).

The other cases were two more cases of regressed hemangiomas, one case of trauma and the last case was solar keratosis of lower lip with scar of previous surgery on his lip mucosa, which all were treated in the same manner.

## RESULTS

The Ahmad-Ali's flap was performed on eight patients. The average patient age was 26.5 years. The average follow-up period was 1.3 years. There were no postoperative complications, e.g., airway obstruction, bleeding, infection, wound disruption, or flap necrosis. Surgical results were satisfactory in all patients, and sufficient lip mobility with adequate bulk was maintained. One patient demonstrated minimal transient lip tightening.

## DISCUSSION

Several techniques have been proposed to deal with defects or irregularities of the vermilion.^1^^-^^6^ Transfer of tissue from the lower lip or the tongue might be required to correct large tissue defects in the vermilion. To resurface the lip in this situation or for complications of transposition flap loss, a tongue flap can be used. Both the upper and lower lips can be reconstructed using tongue musculomucosal flap. The subsequent defects of benign or malignant tumor removal, trauma and secondary deformities due to clefting are the most frequent situations in which this procedure is required .Repair of defects, which are smaller than 1 cm, using free composite tissue grafts from the lower lip are described by Flanagin.^7^

These methods face several challenges: i) Color and texture differences between tongue and vermilion; ii) Long period of interposition of tongue between the teeth; iii) Tenuousness; iv) Limit in the amount of mucosa that can be transferred. Matching color and contour are often improved if centrally based flap of exposed vermilion in cross lip flaps are used. Micro aspirations and gag reflex irritation are also other disadvantages of tongue flaps.^3^^,^^4^

Tongue flaps and free grafts do not often provide a good match for normal vermilion.^3^ Transfer of tissue from the lower lip is usually required in diffuse vermilion defects, e.g., in cases occurring after bilateral cleft lip repairs. This involves Abbe flap and its many variations and pedicled vermilion flaps.^1^^,^^3^^,^^8^^-^^10^ Due to the fact that Abbe and other centrally based cross-lip flaps leave lower lip scars, they are not entirely satisfactory, as each of these require division of the orbicularis oris muscle. Unilateral vermilion flaps do not frequently transfer enough tissue for bilateral defects.^1^

An effective procedure for patients with traumatic or neoplastic defects is vermilion advancement. However, due to the shortcoming of this method in providing vermilion in patients with lip hemangioma lesions that involve skin and mucosa around the defect this method may not be effective. 

The success rate of the proposed method in this paper is very high, close to 100%, and therefore, it is a very justifiable approach. In cases that using flaps from the same lip is not possible, switch flap or cross-lip would be quite feasible. In composite graft method, the success rate is not that high and the chance for graft loss at least in the form of partial loss or color or texture change is very evident.^1^

The most commonly used and oldest cross-lip flap method is Abbe's flap, which is a full thickness composite from skin, muscle and mucosa of one lip to repair the full thickness defect of the cross lip. To the best of our knowledge, no one has ever used a mutual cross-lip flap to cover such a vast defect as well as donor defect coverage at the same time that has been dealt with in this paper. An interesting study in this regard is described by Kawamoto for lip defects. The cross lip transverse vermilion flap is well suited to the repair of major defects of the vermilion.^3^ For reconstruction of large defects of vermilion, the buccal mucosa of the opposite lip can be a donor flap based on the “normal vermilion”, and donor defect can be skin grafted, as described by Gillis and Millard.^11^ Our plastic surgery residents have coined "Ali's flap" for the proposed method in this paper. We prefer to say Ahmad-Ali”s flaps. In patients with lip mucosal complete defects, this procedure is applicable with ease and desired results.

## CONFLICT OF INTEREST

The authors declare no conflict of interest.
